# Tumor Microenvironment Metabolism: A New Checkpoint for Anti-Tumor Immunity

**DOI:** 10.3390/vaccines4040046

**Published:** 2016-12-06

**Authors:** Nicole E. Scharping, Greg M. Delgoffe

**Affiliations:** 1 Tumor Microenvironment Center, University of Pittsburgh Cancer Institute, Pittsburgh, PA 15232, USA; nes63@pitt.edu; 2 Department of Immunology, University of Pittsburgh, Pittsburgh, PA 15213, USA

**Keywords:** T cell, metabolism, immunotherapy, cancer, tumor microenvironment, metabolic checkpoints

## Abstract

When a T cell infiltrates a tumor, it is subjected to a variety of immunosuppressive and regulatory signals in the microenvironment. However, it is becoming increasingly clear that due to the proliferative and energetically-deregulated nature of tumor cells, T cells also operate at a metabolic disadvantage. The nutrient dearth of the tumor microenvironment (TME) creates “metabolic checkpoints” upon infiltrating T cells, impacting their ability to survive, proliferate and function effectively. In this review, we summarize the basics of tumor cell and T cell metabolism and discuss recent advances elucidating the individual metabolic checkpoints exerted on T cells that drive their dysfunction in the TME.

## 1. Introduction

Most of the hallmarks of cancer cells can be attributed to the inability to respond to environmental cues to slow proliferation or induce cell death [1]. These environmental insensitivities are generally due to DNA mutations, usually occurring during an individual’s lifetime and in response to a variety of exogenous genetic insults and endogenous modulatory factors [1]. While the initial neoplastic event is due to these uncorrected DNA mutations, it is widely believed that the progression from neoplasia to malignancy is due in part to a failure of immunosurveillance [2,3,4]. The immune system excels at identifying and eliminating mutated cells, but cancer can evade recognition through a process called “immunoediting”. The immune system puts selective pressure on the tumor cell population, making it advantageous for tumor cells to mutate or alter the production of antigens that might be recognized as foreign by the immune system. Tumor cells also downregulate antigen processing and presentation machinery, rendering them invisible to the immune system. Thus, if cancerous cells keep mutating, they can keep evading immune recognition [5].

Concomitant with cancer cells escaping immune recognition and elimination, cancer cells may begin to utilize additional mechanisms to create an immunosuppressive environment. This can be induced by the recruitment of immunosuppressive cells (myeloid-derived suppressor cells (MDSCs) and regulatory T cells) to create a “wound healing” environment and the generation of tolerogenic signals, such as interleukin-10 (IL-10), transforming growth factor-beta (TGF-β) and extracellular adenosine [6]. Tumor cells can induce T cell dysfunction through direct receptor-ligand interactions, expressing co-inhibitory ligands, such as programmed death-ligand 1 (PD-L1) to inhibit CD8^+^ tumor-infiltrating lymphocytes’ (TIL) function through programmed death-1 (PD-1), a process further enhanced through contact with the immune system [7]. T cell function can also be inhibited by other co-inhibitory “checkpoint” molecules, such as cytotoxic T lymphocyte-associated protein-4 (CTLA-4), T cell immunoglobulin and mucin domain containing-3 (Tim-3), lymphocyte activating gene 3 (Lag3) and T cell immunoreceptor with Ig and ITIM domains (TIGIT), which are upregulated on the surface of T cells after activation and remain highly expressed on T cells in the tumor microenvironment (TME) due to persistent activation signals. Ligation of these co-inhibitory checkpoint molecules results in downregulation of T effector function.

One of the most promising new immunotherapies, generally termed “checkpoint blockade”, utilizes monoclonal antibodies specific to either the co-inhibitory ligand or receptor to block their interaction [8]. Anti-CTLA-4 and anti-PD1/PD-L1 were among the first in clinical trials, showing promising objective clinical responses [9,10]. However, these therapies are only effective in a subset of patients, and the biomarkers of responsiveness to these immunotherapies remain elusive [11]. This likely indicates that immunosuppression from these “immunologic” sources does not account for the whole suppressive microenvironment. In this review, we postulate that tumor cells are also immunosuppressive due to a suppressive metabolic microenvironment characterized by a lack of crucial carbon sources and intermediates needed for T cell function.

## 2. Metabolism in the Tumor Microenvironment

The driving force behind the malignancy and morbidity of cancer is its ability to proliferate unrestrained. While individual cancer cells may be insensitive to growth inhibition, it is not without cost for these cells. Their unrestrained growth requires the cancer cells to utilize aerobic glycolysis (also called the “Warburg effect”, after Otto von Warburg who initially described it) over oxidative metabolism [12,13]. This occurs when cells convert glucose-derived pyruvate into lactic acid, rather than acetyl-CoA to fuel oxidative phosphorylation (OXPHOS). While this glucose fermentation occurs in all cells when oxygen is limiting, most tumors cells acquire a metabolic adaptation to perform glycolysis even in the presence of oxygen [14].

Why glycolysis occurs in cancer cells has been a matter of debate since its discovery [12]. It was originally hypothesized that aerobic glycolysis may occur in cancer cells due to mitochondrial damage, but it is now clear that cancer cells still utilize their mitochondria for oxidative metabolism [15]. Thus, it may seem perplexing why a tumor cell might choose this bioenergetically unfavorable pathway, as biochemical studies show that glycolysis generates eighteen times less ATP per mole of glucose than OXPHOS [16,17]. However, there are other important considerations for the cellular metabolism of tumor cells. First, while glycolysis generates less ATP per mole of glucose compared to OXPHOS, the kinetics of this reaction are considerably different: glycolysis generates ATP nearly a hundred times faster than OXPHOS, such that if a tumor cell could compete for glucose, it could meet its metabolic demands [16,17]. Second, by utilizing aerobic glycolysis, the cancer cell can regenerate the reductive molecule NAD^+^, which is utilized in the initial steps of glycolysis, as well as in other essential metabolic pathways in the cell. Third and most importantly, aerobic glycolysis serves to free a portion of mitochondrial function to perform anabolic metabolism: producing the lipids, amino acids and nucleotides needed to generate daughter cells [14,18]. Due to the high metabolic demands of these highly proliferative cells, cancer cells utilize aerobic glycolysis and use up essential metabolites in the surrounding environment, secreting lactic acid as metabolic waste of the glycolytic pathway [14]. As a result, this microenvironment has low glucose, low oxygen and a low pH [17,19,20,21]. These crucial metabolites, especially oxygen, are replenished poorly due to dysregulated angiogenesis caused by the tumor [22]. Low oxygen levels also help induce a glycolytic program by increasing HIF-1α expression [23]. HIF-1α turns on gene expression programs to directly inhibit OXPHOS, further enforcing tumor cell aerobic glycolysis [24].

**Figure 1:**
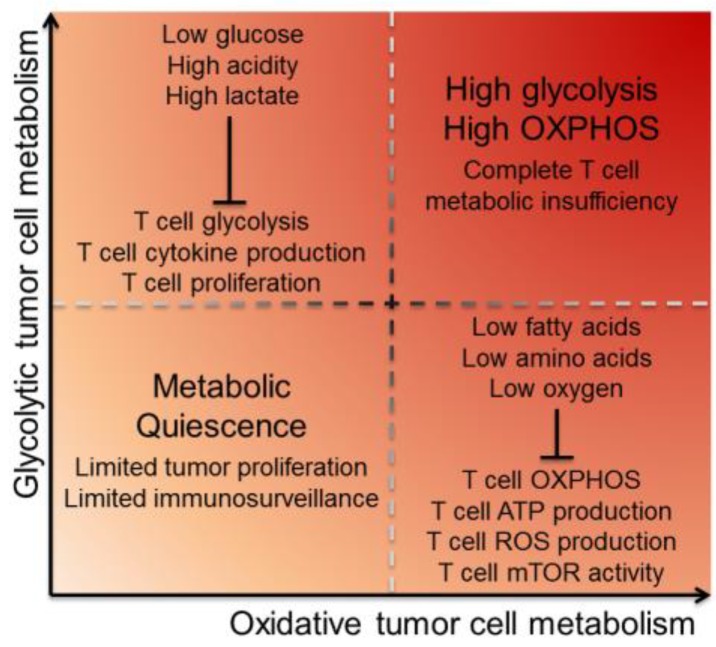
Tumor cell energetics dictate the metabolic landscape of the TME. Tumor cells utilize glycolysis and oxidative phosphorylation metabolism, causing depletion of particular environmental metabolites and inhibiting TIL in different ways. Glycolytic tumors primarily utilize glycolysis for energy, creating a TME that has low glucose and high acidity and lactate. This inhibits T cell function by limiting fuel for T cell glycolysis, as well as inhibiting T cell cytokine production and proliferation. Oxidative tumors primarily utilize OXPHOS and their mitochondria for energy, creating a TME that has low oxygen, low fatty acids and low amino acids. This inhibits T cell function by limiting oxygen for T cell OXPHOS, as well as inhibiting T cell mitochondria from producing ATP and ROS. Low ATP production has the downstream consequence of inhibiting mTOR activity. Tumor cells that are both glycolytic and oxidative inflict complete metabolic insufficiency on T cells, while quiescent tumors with minimal metabolic demands can better hide from immunosurveillance via reduced proliferation. Abbreviations: tumor microenvironment (TME), tumor-infiltrating lymphocyte (TIL), oxidative phosphorylation (OXPHOS), reactive oxygen species (ROS), and mechanistic target of rapamycin (mTOR).

The altered metabolism of tumor cells was posited by Warburg to be not only a characteristic of malignancy, but even causative of the malignant phenotype [12]. While the last several decades have explored exactly why and how developing cancer undergoes this type of metabolic transformation, it is clear that this emerging hallmark of cancer does not only impact the cancer cells themselves [1]. This hunger in cancer cells for available nutrients to fuel their unrestrained proliferation creates a bioenergetic sink for any cell that enters the tumor microenvironment. It is now understood that a lack of metabolites and oxygen, as well as high intratumoral acidity, act as additional immunosuppressive mechanisms the tumor exploits to prevent infiltrating T cells from functioning efficiently (Figure 1).

## 3. Metabolic Regulation of T Cells

Recent studies have demonstrated the essential role of metabolism for immune cells, beyond simply growth or death decisions. The link between fuel source and metabolic program and how these impact immune cell phenotype and function are an active area of research [25]. One of the first contributions to the immunometabolism field was looking at the relative contributions of glycolysis and OXPHOS during lymphocyte activation [26]. It was noted that when lymphocytes were activated via receptor crosslinking with phytohemagglutinin, the lymphocytes (like tumor cells) also underwent aerobic glycolysis. Later studies revealed that T cells use both glycolysis and OXPHOS to generate ATP, and both pathways are required for in vitro proliferation [27]. As T cells also undergo aerobic glycolysis upon activation, they have high energy demands from their environment in order to proliferate and function. To better understand the basics of T cell metabolism, the following section will review when T cells change their metabolic demands, as well as how environmental nutrient sensing can change the fate of T cells. With this understanding, we will then see how energy sensing and utilization becomes dysfunctional in the TME when T cells need to compete with tumor cells for glucose and other nutrients.

### 3.1. T Cells Change Their Metabolic Demands upon Activation

Of all immune cells, T cells in particular have become a research focus, as the progression through activation comes with distinct metabolic changes [28]. Naive T cells, which are required to be quiescent for a lifetime, have low metabolic demands. The maintenance of this quiescent state requires minimal OXPHOS, dividing only to maintain the clonal repertoire throughout life. The pro-survival cytokine interleukin-7 (IL-7) is necessary for the maintenance of these quiescent cells [29]. IL-7 is important for the uptake of glucose through glucose transporter 1 (Glut1) for the maintenance of ATP levels [30]. Thus, these quiescent T cells maintain minimal metabolic demands until activated, preserving fuel in the environment for those T cells that have entered their effector phase.

When a T cell encounters its antigen, its first task is to begin cellular growth and to prepare for cell division. Since antigen-specific naive T cells are so few in numbers, it is important to proliferate quickly to generate sufficient numbers of effector cells; but this proliferation has high metabolic demands. Thus, like highly proliferative tumor cells, activated T cells utilize glucose as a primary fuel source [31]. Glut1 is upregulated upon T cell activation, through T cell receptor (TCR) and CD28-induced serine/threonine kinase Akt activation, allowing activated T cells to increase their glucose uptake eighteen-fold higher than naive T cells [32]. This allows cells to use aerobic glycolysis for anabolic cell growth [30]. Failure to upregulate metabolic pathways upon T cell activation leads to a hyporesponsive phenotype much like clonal anergy, a state in which even a full antigenic stimulation cannot induce a T cell response [33]. Similar to an anergic T cell, T cells that have been activated in nutrient-poor conditions will become unresponsive to future stimulation. This finding highlights the important link between metabolism and T cell function: starving a T cell can have lasting effects on its ability to perform in the future, even in nutrient-rich conditions.

While adopting aerobic glycolysis can keep up with ATP demands, regenerate NAD^+^ and promote anabolic pathways for mitochondrially-derived substrates, recent studies have also revealed that glycolysis may contribute to effector functions directly. It has long been known that glycolytic enzymes can “moonlight” as RNA-binding proteins when not performing enzymatic reactions [34]. Indeed, T cell-derived glyceraldehyde 3-phosphate dehydrogenase (GAPDH) was shown to bind interferon gamma (*Ifng*) mRNA in resting, non-glycolytic T cells, suggesting that glycolysis promotes cytokine secretion, in part, by relieving post-transcriptional repression of cytokine messages [27].

T cells continue to utilize aerobic glycolysis during the effector phase of their activation, but switch back to primarily utilizing OXPHOS as they become memory cells [35]. The formation of memory T cells leads to a larger mitochondrial reserve than naive T cells, termed spare respiratory capacity [35]. The gamma-chain cytokine interleukin-15 (IL-15), important for forming long-lasting memory T cells, also increases mitochondrial biogenesis and the expression of the mitochondrial enzyme carnitine palmitoyltransferase 1a (CPT1a), a rate-limiting enzyme in fatty acid oxidation [36]. As such, it is thought that the increase in memory T cell mitochondria primes the cells for a quick recall response with a bioenergetic advantage over naive T cells. Memory T cells in a recall response can also switch to glycolysis in an Akt-dependent manner, but have higher glycolytic flux and increased upregulation of glycolytic enzymes, such as GAPDH, than do naive T cells [37]. However, antigen-experienced T cells also have additional metabolic demands, as evidenced by the fact that memory T cells also rely on fatty acid oxidation. For instance, when CD8 T cells lack TNF receptor associated factor 6 (TRAF6), a signaling molecule downstream of the tumor necrosis factor (TNF) cytokine receptor superfamily, they are unable to form memory after immunization [38]. TRAF6-deficient T cells show repression of fatty acid metabolism genes, leading to defective lipid oxidation. Thus, memory T cells may require a variety of metabolites to fuel their increased mitochondria to carry out their recall responses more effectively.

The metabolism of T cells is tied directly to proliferation, cytokine production and cytolytic function: essential functions of an effector T cell. While many of these studies have utilized inhibitors or genetic targeting to determine the contributions of various metabolic pathways to T cell function, a pair of recent studies has revealed that these pathways are dynamically regulated even during the generation of a “typical” T cell response [39,40]. The model for the generation of memory hypothesizes that T cells have both short-lived effector and memory potential. However, upon contact with antigen-presenting cell (APC)-bound peptide-major histocompatibility complex (MHC), the T cell will grow and then divide in an asymmetric manner. The proximal cell to the APC receives the lion’s share of signaling molecules and activation signals and becomes fated to be a short-lived, effector cell, while the distal cell is less proliferative in subsequent divisions and inherits more memory potential [41]. Interestingly, the metabolic demands of a T cell can also be asymmetrically inherited upon activation [39,40]. Proximal daughter cells have more Myc and mechanistic target of rapamycin (mTOR) activity, resulting in part due to asymmetric partitioning of lysosomes and amino acid transporters. As both Myc and mTOR are important factors for cell proliferation, differentiation and metabolism, differences in these metabolic programs lead to distinct cell fates for the dividing daughter cells.

### 3.2. T Cells Utilize Nutrient Sensing to Dictate Their Differentiation

It is clear that metabolite availability and changes in metabolic pathways can have major effects on T cell function, but this is largely unsurprising since energy is required to perform any complex cellular task. However, unlike other renewable cell types present in the body, clonal diversity must be preserved in T cells. This creates a need for reliance upon nutrient sensing. It would not be favorable for a T cell to engage an effector response if it could not metabolically support the expansion; subsequent cell death might eliminate that clone. T cells thus have conscripted the nutrient-sensing machinery to make more complex fate determinations, rather than growth-or-death decisions.

A key molecule in the nutrient sensing machinery is mTOR. mTOR integrates environmental signals, such as amino acid and oxygen availability, intracellular ATP concentration, extracellular growth/survival signals, and cellular activation status. These signals are then utilized to induce changes in cell size, proliferation, metabolism and survival to respond to extracellular signals and environmental status [42]. The importance of mTOR in T cells was discovered by its inhibition by rapamycin, a macrolide antibiotic that is a potent immunosuppressant [43]. Rapamycin was first found to induce anergy in T cells [44]. Once rapamycin was determined to inhibit mTOR, the signaling molecule’s role in T cell activation and differentiation was investigated.

mTOR was shown to be the signaling molecule responsible for “full” T cell activation upon TCR engagement in T cell clones. Further activated and anergic T cells could be distinguished by mTOR-induced metabolic machinery, which provided evidence for metabolism’s critical role in T cell functionality [45]. Further exploration of the role of mTOR found that it is essential for T cell lineage commitment [46]. CD4^+^ T cells lacking mTOR differentiate into regulatory T cells over Type 1 T helper (Th1), Th2 or Th17 cells. It is interesting to note the lack of mTOR results in regulatory T cells, since it could be interpreted as the lack of metabolic cues from a T cell’s environment that defaults to a regulatory phenotype and that T cell anergy and regulation are very similar from a metabolic standpoint. CD8^+^ T cells are also dependent on mTOR signaling; low-dose mTOR inhibition increases memory T cell precursors and accelerates memory T cell differentiation, somewhat paradoxically as mTOR is used clinically as an immunosuppressant [47]. As CD8^+^ memory relies more on OXPHOS than glycolysis and mTOR activation is associated with glycolysis and AKT activation, low-level mTOR signaling in CD8 T cells may bias their metabolism towards that which supports a memory phenotype. 

mTOR does not signal by itself; rather, it exists as a larger signaling complex with other molecules. Specific T cell-conditional deletion of mTOR complex 1 (mTORC1) and mTORC2 revealed that these complexes play distinct roles in directing T cell fate. When mTORC1-dependent Ras homolog enriched in brain (Rheb) was deleted from CD4^+^ T cells, they failed to differentiate into Th1 or Th17 T cells, but Th2 differentiation was intact [48]. Additionally, when mTORC2 signaling was conversely deleted, CD4^+^ T cells failed to differentiate into Th2 T cells, but Th1 and Th17 differentiation was intact. The opposing roles of mTORC1 and 2 in T cell fate determination highlight a T cell’s ability to make widely different fate decisions based on subtle metabolic environmental differences. This allows for T cell plasticity and the ability to respond complexly to environmental changes. mTORC1 and 2 were also found to have specific roles in generating effector and memory CD8^+^ T cell subsets. Constitutively-active mTORC1 generates glycolytic effector T cells unable to enter the memory phase, while mTORC1-deficient effector T cells failed to activate an effector response, but maintained memory T cell characteristics [49]. The control of T cell fate by mTORC1 and mTORC2 supports the idea that the T cells have cell-intrinsic potential to be either effector or memory upon activation, rather than effector T cells differentiating to memory as a progression of activation. The ability of mTOR to sense the environmental state thus impacts T cell activation and differentiation; nutrient availability can have a long-term impact on T cell fate.

Outside of mTOR, T cells use other metabolic signaling molecules to sense and respond to their environment, namely Myc and 5′ AMP-activated protein kinase (AMPK). Myc is essential for glycolytic reprogramming, with deletion of Myc inhibiting activation-induced glycolysis and glutaminolysis [50]. AMPK senses nutrient deprivation and can limit mTOR activity in low glucose environments [51]. AMPK also impacts T cell differentiation; AMPKα1 knockout T cells are unable to form memory and display defects in CD4^+^ Th1 and Th17 differentiation [51,52].

Additional transcription factors can also play a role in how T cells respond to environmental metabolic cues promoting cellular proliferation, differentiation, or homeostasis. Sterol regulatory-element binding proteins (SREBPs) are a family of transcription factors that control cholesterol synthesis and uptake. SREBP family members are upregulated upon T cell activation and are essential to proliferating CD8^+^ T cells, required for the increased lipid membrane synthesis needed to generate daughter cells [53]. Peroxisome proliferator-activated receptors (PPARs), the family of nuclear receptor transcription factors also involved in lipid metabolism, have also been shown to play a role in T cell immunoregulation [54]. PPARγ has specifically been shown to be a negative regulator of T cell activation, regulating interleukin-2 (IL-2) production in activated T cells, and the loss of PPARγ in T cells contributes to autoimmunity [55,56]. Transcription factor EB (TFEB), a regulator of lysosomal biogenesis, has been shown to be negatively regulated by mTORC1 [57]. When intracellular metabolites are high, TFEB is retained in the cytosol, but when nutrients are needed, TFEB disassociates from mTORC1 and translocates to the nucleus to increase lysosome biogenesis. Lysosomes are important for mTORC1 activation and further downstream signaling, but the effects of the loss of TFEB in T cells are still unknown. However, transcription factor A mitochondrial (TFAM) has also been shown to play a role in T cell lysosomal function [58]. Deletion of TFAM in T cells impairs lysosomal function and promotes proinflammatory T cell differentiation, linking T cell homeostasis with differentiation. For mitochondrial function, Yin Yang 1 (YY1) acts as a mediator between mTOR and mitochondrial biogenesis; mTOR activates YY1 to promote mitochondrial biogenesis via transcriptional coactivator PPAR gamma coactivator 1 alpha (PGC1α) [59]. These families of transcription factors allow T cells to integrate environmental metabolite signals, initiating fine-tuned gene programs associated with cell proliferation, differentiation, and homeostasis.

As described above, T cells rely on metabolism to both become activated and make complex fate determinations. However, these decisions are often carried out in environments where T cells have adequate metabolites to carry out these functions. When the microenvironment cannot metabolically support T cell expansion, the signals to grow and differentiate can be misinterpreted, resulting in T cell dysfunction.

## 4. Metabolism of T Cells in the Tumor Microenvironment

**Figure 2:**
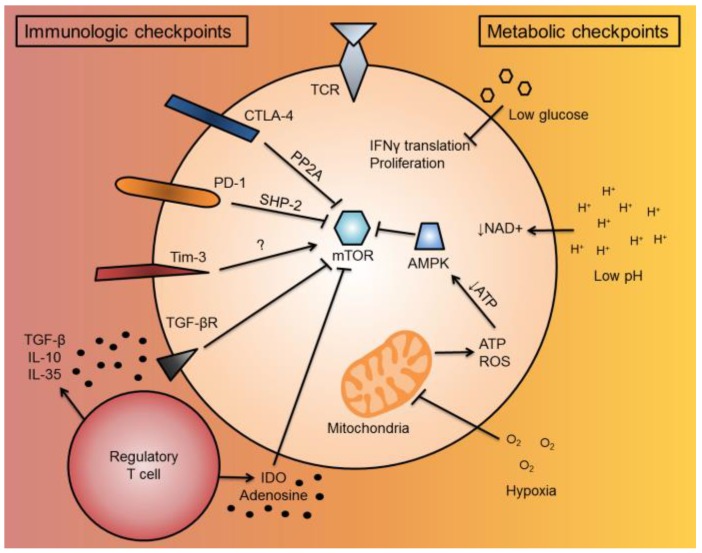
T cell immunologic and metabolic checkpoints in the tumor microenvironment. When a T cell enters the TME, it is subjected to a variety of immunosuppressive and metabolic signals, termed “immunologic and metabolic checkpoints”, which have overlapping functionalities to suppress T cell function. When a T cell becomes activated, it upregulates co-inhibitory molecules, such as CTLA-4 and PD-1, to inhibit further activation. These cell-intrinsic co-inhibitory molecules inhibit mTOR function through PP2A or SHP-2 signaling, respectively. Tim-3, another co-inhibitory molecule, can activate mTOR through unknown mechanisms. For cell-extrinsic immunologic checkpoints, immunosuppressive cells in the TME, such as regulatory T cells and MDSCs, can secrete or convert soluble factors, such as TGF-β, IL-10, IL-35, IDO, or adenosine, which suppress T cell activity. TGF-β can suppress T cell activity by suppressing mTOR through TGF-βR signaling. IDO also can suppress mTOR. For metabolic checkpoints, low glucose has been shown to inhibit IFNγ cytokine translation and T cell proliferation. The low pH of the TME prevents the regeneration of NAD^+^, important as a reducing equivalent to drive the mitochondrial TCA cycle forward to generate ATP. TME hypoxia can also inhibit the mitochondria by limiting electron acceptors of ATP synthase. This can decrease T cell ROS, as well as decrease cellular ATP levels. Low cellular ATP activates AMPK, which can further inhibit mTOR activity. Abbreviations include tumor microenvironment (TME), cytotoxic T lymphocyte-associated protein-4 (CTLA-4), programmed death-1 (PD-1), mechanistic target of rapamycin (mTOR), protein phosphatase 2A (PP2A), SH2-domain containing tyrosine phosphatase 2 (SHP-2), T cell immunoglobulin and mucin domain containing-3 (Tim-3), myeloid-derived suppressor cells (MDSCs), transforming growth factor-beta (TGF-β), interleukin-10 (IL-10), interleukin-36 (IL-35), indoleamine 2,3-dioxygenase (IDO), interferon gamma (IFNγ), tricarboxylic acid (TCA), reactive oxygen species (ROS), and 5′ AMP-activated protein kinase (AMPK).

When T cells infiltrate the TME, they are subjected to immunosuppressive signals and low metabolites. These signals impact T cell function in the form of immunologic and metabolic checkpoints (Figure 2). In this section, we will discuss immunologic and metabolic checkpoints and how they overlap to suppress effector function in the TME.

### 4.1. T Cell Co-Inhibitory Signaling and Metabolism

Classical immunosuppressive mechanisms in the TME can overlap with the more recently elucidated metabolic checkpoints. T cell co-inhibitory molecules, found on CD8^+^ T cells that enter the TME, have signaling pathways that intersect with metabolic signaling. The co-inhibitory molecule CTLA-4, which gets upregulated upon T cell activation and competes with CD28 for B7 ligands, can intersect with mTOR via protein phosphatase 2A (PP2A) [60]. PP2A, a regulatory phosphatase involved with cell cycle regulation, is recruited to the cytoplasmic tail of CTLA-4 and can inhibit mTOR activity by dephosphorylating and inactivating upstream Akt [60,61,62]. The co-inhibitory molecule PD-1 can also inhibit mTOR via SH2-domain containing tyrosine phosphatase 2 (SHP-2) signaling [63,64,65]. SHP-2 is recruited to the immunoreceptor tyrosine-based switch motif (ITSM) sequence in the PD-1 cytoplasmic tail, and SHP-2 has been shown to inhibit phosphoinositide 3-kinase (PI3K) activity, thereby inhibiting downstream Akt and mTOR activity. Forkhead box protein o1 (Foxo1), a transcription factor downstream of Akt and mTOR signaling, also intersects with co-inhibitory signaling [66]. Impaired Akt and mTOR signaling due to chronic viral infection enhances Foxo1 activity, thereby increasing the expression of PD-1 on T cells. Additionally, Tim-3, which is found upregulated on exhausted T cells, has been implicated in upregulating ribosomal protein S6, downstream of the PI3K/Akt/mTOR pathway [67]. Rather than inhibiting mTOR, Tim-3 could be promoting it, increasing glycolysis and other metabolic functions.

T cells upregulate co-inhibitory molecules to prevent further activation of already activated cells [8]. This prevents the pathological effects of unrestrained activation and potential autoimmune or autoinflammatory tissue damage. It makes sense that these co-inhibitory molecules (CTLA-4 and PD-1) evolved to also inhibit the metabolic phenotype of activation. However, the ligand used to signal through Tim-3 in the tumor microenvironment and the consequent downstream signaling pathways are still unclear. However, the idea that Tim-3 might increase mTOR function may help to discover the true function of Tim-3 on activated T cells.

### 4.2. Low Glucose and T Cell Function

The importance of glucose for T cell function was first determined in in vitro assays. When T cells have limited glucose, they decrease their glycolytic flux through a decrease in Akt activity and can activate proapoptotic B cell lymphoma-2 (Bcl-2) family members to induce apoptosis [68,69,70]. Recent work has begun to explore in-depth how the metabolite-deficient TME impacts the effector function of T cells. Several recent studies in tumor-T cell immunometabolism have focused on understanding how low glucose availability impacts T cell functionality in the TME [71,72,73]. Rendering tumor cells more glycolytic by increasing tumor cells’ glucose uptake makes CD8^+^ T cells less capable of controlling tumor growth [71]. It was also shown that tumor cell-specific checkpoint blockade reduces tumor cell glucose uptake, restoring glucose to the TME, which may be one reason for the success of co-inhibitory checkpoint blockade as an immunotherapeutic [71]. In addition, another recent study has shown that the glycolytic metabolite phosphoenolpyruvate is important for sustaining calcium-mediated nuclear factor of activated T cells (NFAT) signaling to enable conventional CD4^+^ T cell function in the tumor [72]. The low glucose TME has also been shown to decrease methyltransferase enhancer of zeste homolog 2 (EZH2) expression in T cells in the setting of ovarian cancer [73]. Low EZH2 expression lead to T cell’s decreased ability to perform glycolysis and effector functions and decreased T cell survival. Therefore, glucose is an essential metabolite for T cell function, but its limited availability in the TME contributes to metabolically-induced T cell dysfunction.

### 4.3. Hypoxia and Tumor-Infiltrating T Cell Function

Oxygen availability is low in the TME due to both poor vascularization of the tumor and the high oxygen consumption of proliferative tumor cells [21,22]. Decreased oxygen tension is a classic hallmark of the tumor microenvironment, but its effects of hypoxia on T cell function are still nebulous [1,74]. Much of our understanding of hypoxia’s effect on T cells is derived from genetic manipulation of the hypoxia sensing machinery: prolyl hydroxylase domain-containing proteins (PHD), Von Hippel-Lindau (VHL) and hypoxia inducible factor 1 alpha (HIF1α) [74]. HIF1α is stabilized in hypoxia, but it is also stabilized upon T cell activation, due in part to Myc upregulation and consequent glycolytic reprogramming [50]. Thus, it is difficult to interpret how the deletion or overexpression of HIF1α might actually represent a T cell’s response to hypoxia in vivo. Nevertheless, in several different types of experimental systems, low oxygen seems to decrease T cell proliferation, with variable effects on cell survival and function [75]. Oxygen availability and its impact on infiltrating CD8^+^ T cell function have also begun to be explored by giving supplemental oxygen to tumor-bearing mice to decrease TME hypoxia [76]. Decreasing hypoxia through respiratory hyperoxia led to increased T cell infiltration, increased proinflammatory cytokines and improved tumor regression and survival in mice. Overall, oxygen is an important metabolite needed both for T cell and tumor cell function and is robustly competed for in the TME.

### 4.4. TME Metabolites and Tumor-Infiltrating T Cell Function

The TME has low or altered metabolites compared to different environments in the body. The TME has low availability of many amino acids, especially glutamine, due to its use as a primary fuel for tumor cells [77]. Because glutamine is an important amino acid for T cells and a lack of glutamine prevents T cell activation and differentiation, the low glutamine TME is detrimental for T cell function [78,79]. The amino acid tryptophan is also low in the TME, due to the tryptophan-metabolizing enzyme indoleamine 2,3-dioxygenase (IDO) expressed by tumor cells and regulatory immune cells [80]. Both the loss of tryptophan from the environment and the increase of the tryptophan catabolite kynurenine are immunosuppressive. The amino acid arginine is also low in the TME, due to its consumption by tumor cells and by myeloid cell arginase-mediated depletion, and arginine is required in activated and proliferating T cells [81,82]. Lactic acid, the byproduct of deregulated tumor cell glycolysis, is extraordinarily immunosuppressive, inhibiting T cell function and cytokine production through both pH-dependent changes, as well as loss of cytosolic NAD^+^ regeneration [83]. In addition, extracellular adenosine has been shown to be immunosuppressive. Extracellular ATP, released from dying cells, can be metabolized by CD39 and CD73 expressed on the surface of regulatory T cells, MDSCs and tumor cells [84]. This adenosine binds to the adenosine A_2A_ receptor on the surface of T cells to suppress T cell activity and induce regulatory T cells. Thus, the TME has many metabolic deficiencies, every one of them detrimental on its own for T cell function. T cell activation may be crippled in the TME due to this “perfect storm” of metabolically-suppressive signals, acting along many axes to restrain T cell function.

### 4.5. Mitochondria and Tumor-Infiltrating T Cell Function

Mitochondria are more than the “powerhouse” of the cell: they are biosynthetic factories, used to generate the material for daughter cells, providing numbers for a T cell clonal army [18]. Mitochondria also are essential for T cell effector function, especially to generate reactive oxygen species (ROS) for T cell cytolytic activity [85]. Recently, even the morphology of mitochondria has been linked to T cell fate. Activated effector T cells undergo dramatic mitochondrial remodeling, forming punctate mitochondria caused by fission, while memory T cells are characterized by a fused network of mitochondria under the control of mitochondrial fusion protein optic atrophy 1 (Opa1) [86]. These mitochondrial fusion networks have superior oxidative function, an important hallmark of T cell memory, and function better in solid tumors. It may be that these mitochondrial morphologies impact T cell function directly, and future studies in mitochondrial and metabolic dynamics will identify how these crucial processes are dynamically regulated and reprogrammed during the life of the T cell. However, these processes can be critically regulated in response to cancer.

**Figure 3:**
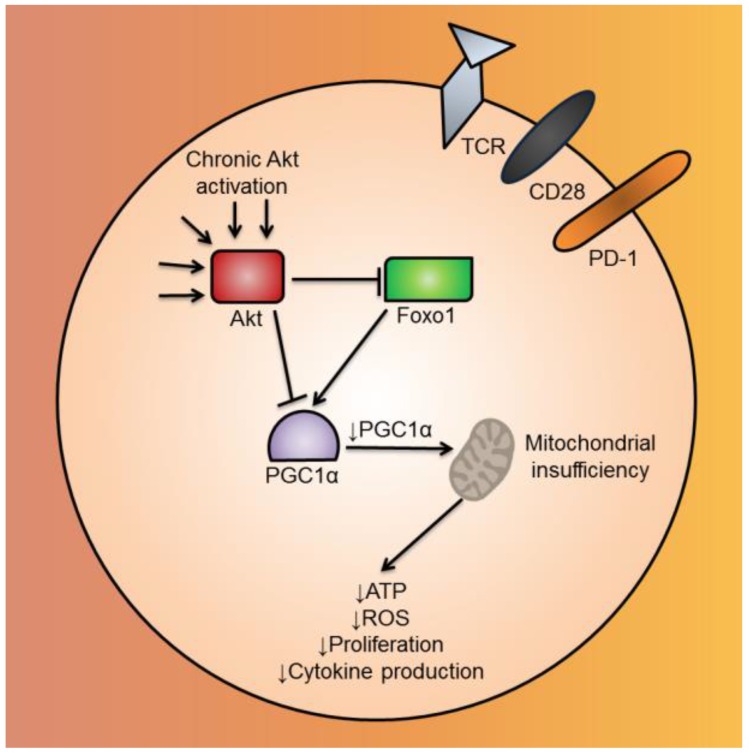
Mitochondrial biogenesis is repressed by Akt-mediated repression of PGC1α in CD8^+^ TIL. CD8^+^ TIL in the TME have chronically-activated Akt. Akt, either acting directly or indirectly via Foxo1, inhibits the mitochondrial biogenesis transcriptional coactivator, PGC1α. Repressed mitochondrial biogenesis leads to mitochondrial insufficiency, causing the CD8^+^ TIL to have functional defects, such as decreased ATP, ROS, cytokine production and proliferation. Abbreviations include tumor-infiltrating lymphocyte (TIL), tumor microenvironment (TME), Forkhead box protein o1 (Foxo1), PPAR gamma coactivator 1 alpha (PGC1α), and reactive oxygen species (ROS).

We have recently shown that T cells lose mitochondrial activity and mass upon entering the TME, a process that correlates with upregulation of co-inhibitory checkpoint molecules [87] (Figure 3). This loss in mitochondrial mass is caused by decreased mitochondrial biogenesis, due in part to repression of the transcriptional co-activator PGC1α. Chronic Akt signaling was found to drive down PGC1α in tumor T cells, and PGC1α levels could be partially restored with Akt inhibition, consistent with the finding that Akt inhibition promotes the expansion of quantitatively and qualitatively better TIL for adoptive cell therapy [88]. Enforcing PGC1α expression via genetic reprogramming also increased mitochondrial fitness in TIL, improving T cell functionality and decreasing tumor burden when adoptively transferred into a melanoma mouse model [87]. Similar mitochondrial deficiencies have been observed in T cells in chronic viral infection, suggesting that repressed PGC1α acts as a driving factor for decreased mitochondrial activity [89]. Thus, improving mitochondrial quantity by programming mitochondrial biogenesis or improving the quality by promoting fusion and morphologic changes may serve to endow T cells with greater persistence and function in the nutrient dearth of the TME.

## 5. Conclusions

The success of several immunotherapeutic approaches in cancer has generated incredible interest in tumor immunology, promoting the search for the next big checkpoint that might surpass PD-1. We would argue that along with the immune checkpoints that inhibit T cell responses in the TME, there are a variety of metabolic checkpoints that negatively impact TIL function. From low extracellular metabolites to inhibitory metabolic signaling, the TME is an environment that by its basic nature prevents T cells from functioning adequately or killing their targets. While metabolic deregulation is a relatively common phenotype of cancer cells, it is clear that there is considerable heterogeneity between patients and even intratumorally, suggesting that some patients may harbor more “metabolically aggressive” tumors than others. Thus, understanding the metabolic qualities of a patient’s tumor could provide clinicians with biomarkers to better understand the specific metabolically-inhibitory nature of a cancer. Furthermore, understanding the precise energetic defects in TIL and the effect of the microenvironment on these defects has the promise to not only synergize to improve existing immunotherapies, but potentially provide novel avenues to re-invigorate endogenous T cells by directly relieving these potent metabolic checkpoints.
